# Differentiating tuberculous pleurisy from pulmonary tuberculosis using mNGS: a multicenter cohort analysis

**DOI:** 10.3389/fcimb.2026.1772516

**Published:** 2026-08-06

**Authors:** Yangmiao Zhang, Ziyi Li, Xiaoke Sun, Chuwen Wang, Zujiang Yu

**Affiliations:** 1 State Key Laboratory of Metabolic Dysregulation & Prevention and Treatment of Esophageal Cancer, Tianjian Laboratory of Advanced Biomedical Sciences, School of Convergence Medicine, Zhengzhou University, Zhengzhou, China; 2 Gene Hospital of Henan Province, The First Affiliated Hospital of Zhengzhou University, Zhengzhou, China; 3 Department of Tuberculosis, The Affiliated Chest Hospital of Zhengzhou University, Zhengzhou, China; 4 Department of Infectious Diseases, The First Affiliated Hospital of Ningbo University, Ningbo, China; 5 Department of Infectious Diseases, The First Affiliated Hospital of Zhengzhou University, Zhengzhou, China

**Keywords:** clinical prediction model, diagnostic differentiation, metagenomic next-generation sequencing, pulmonary tuberculosis, tuberculous pleurisy

## Abstract

**Background:**

Tuberculous pleurisy (TBP), a major extrapulmonary form of tuberculosis, is characterized by a paucibacillary state that makes diagnosis challenging. Metagenomic next-generation sequencing (mNGS) has emerged as a promising approach for MTB detection; however, its discriminatory value between TBP and pulmonary tuberculosis (PTB) among mNGS-confirmed cases, and its integration with clinical features for differential diagnosis, remain insufficiently defined.

**Methods:**

This multicenter retrospective cohort included hospitalized patients with MTB-positive mNGS results from January 2020 to January 2025. As only mNGS-positive cases were included, overall mNGS diagnostic sensitivity cannot be estimated. Twelve TBP patients were matched 1:2 with twenty-four PTB patients by age and sex; patients with immunosuppressive conditions were excluded prior to matching. Clinical, laboratory, mNGS, and conventional TB test data were collected. Logistic regression and ROC analyses were performed.

**Results:**

Conventional tests showed limited sensitivity in TBP despite universal mNGS positivity. MTB read counts were similar between groups (median 1976.5 vs. 990.0, P = 0.920). Pleural-derived specimens predominated in TBP (41.7% vs. 4.2%, P = 0.007). CRP demonstrated the highest individual discriminatory value (AUC = 0.658, P = 0.131), though no single predictor reached significance. A combined model (cough, fever, CRP, WBC) showed modest non-significant improvement (AUC = 0.722, overall P = 0.359; sensitivity 66.7%, specificity 83.3%). Given EPV ≈ 3, all findings are exploratory only. No significant prognostic predictors were identified in TBP; a non-significant trend toward lower lymphocyte counts was observed in patients with unfavorable outcomes (0.60 vs. 1.10 ×10^9^/L, P = 0.115).

**Conclusions:**

Among mNGS-confirmed cases, MTB read counts were comparable between TBP and PTB. No single parameter reliably distinguished the two; a combined clinical model showed modest improvement but requires prospective validation in larger cohorts. Integrating mNGS with systematic clinical evaluation remains essential for accurate TB diagnosis.

## Introduction

Tuberculosis (TB) remains a major global public health challenge, and pulmonary tuberculosis (PTB) is the most common clinical form. Tuberculous pleurisy (TBP), one of the most frequent extrapulmonary manifestations, is typically characterized by a paucibacillary state and nonspecific clinical features (Chan and Lee, 2024). Patients often present with fever, pleuritic chest pain, or dyspnea, which may be indistinguishable from malignant, parapneumonic, or other inflammatory pleural effusions. This overlap frequently leads to diagnostic delays and misclassification in clinical practice (McNally et al., 2023).

Conventional diagnostic methods for TBP—including smear microscopy, culture, and nucleic acid amplification tests—have limited sensitivity due to the inherently low bacterial burden in pleural fluid and pleural tissue. Among NAATs, loop-mediated isothermal amplification (TB-LAMP) has been included in the WHO’s first Essential Diagnostics List and is recommended for active pulmonary TB diagnosis in primary care settings; however, its performance in paucibacillary extrapulmonary specimens such as pleural fluid remains limited (Liu, 2025). Although pleural biomarkers such as adenosine deaminase or interferon-γ can support the diagnosis, they lack specificity and may be confounded by malignancy, autoimmune disease, or other infections (Porcel, 2016). As a result, TBP is frequently underdiagnosed or diagnosed late, and reliable tools to distinguish TBP from PTB and other causes of pleural effusion are still needed.

Metagenomic next-generation sequencing (mNGS) has emerged as a powerful, non-targeted approach for pathogen detection. Recent studies and meta-analyses have shown that mNGS can achieve high sensitivity and specificity for Mycobacterium tuberculosis (MTB) across diverse specimen types, including bronchoalveolar lavage fluid (BALF), blood, and pleural effusion (You et al., 2024). However, most existing work has focused on overall diagnostic yield or early detection of TB, often in mixed or predominantly pulmonary populations; direct comparison of mNGS performance and quantitative metrics between TBP and PTB specifically among mNGS-confirmed cases remains limited, and the discriminatory value of mNGS-derived parameters in this context has not been systematically evaluated (Li et al., 2023; Xu et al., 2023; Lai et al., 2025).

Despite the expanding use of mNGS, several important knowledge gaps remain. First, current studies mainly evaluate overall diagnostic performance, whereas direct head-to-head comparisons of TBP and PTB among mNGS-positive patients are extremely limited. The true differences in mNGS-derived metrics—such as read counts or genomic coverage—and their discriminatory significance therefore remain unclear. Second, the impact of specimen type on mNGS interpretation has not been systematically assessed, even though pleural samples and pulmonary specimens differ markedly in biological characteristics and bacterial burden. Third, no prior study has integrated mNGS indicators with routine clinical features to build a practical model for distinguishing TBP from PTB, leaving clinicians without guidance on how to interpret mNGS in complex real-world settings. Finally, the prognostic value of mNGS parameters in TBP has rarely been investigated, and potential host or laboratory markers associated with short-term outcomes remain unexplored.

Beyond diagnosis, TBP patients frequently exhibit heterogeneous clinical courses and treatment responses. Some experience rapid clinical improvement, whereas others have prolonged symptoms, recurrent effusions, or even unfavorable outcomes despite anti-TB therapy. Prognostic markers for TBP are poorly defined, and it is unclear whether mNGS-derived parameters—such as MTB read counts or genomic coverage—can serve as surrogates for pathogen burden or predictors of outcome.

Therefore, in this multicenter retrospective cohort study, we compared TBP and PTB exclusively among hospitalized adults with mNGS-confirmed MTB infection, acknowledging that this design limits generalizability to the broader TB-suspected population. Our aims were threefold: (1) to exploratorily assess the discriminatory value of mNGS-derived metrics and routine clinical indicators for differentiating TBP from PTB within the mNGS-positive population; (2) to characterize the clinical, laboratory, and specimen-related features associated with TBP in this context; and (3) to identify potential prognostic signals among TBP patients as a basis for future hypothesis-driven research. Given the small sample size, all findings should be interpreted as preliminary and hypothesis-generating rather than definitive.

## Methods

### Study design and participants

This multi-center retrospective cohort study included hospitalized patients who underwent metagenomic next-generation sequencing (mNGS) and tested positive for Mycobacterium tuberculosis (MTB) between January 2020 and January 2025. Data were collected from The First Affiliated Hospital of Zhengzhou University, The Affiliated Chest Hospital of Zhengzhou University, and The First Affiliated Hospital of Ningbo University.

The inclusion criteria were: (1) age ≥18 years; (2) MTB-positive results by mNGS; and (3) complete clinical and follow-up data. Exclusion criteria were: (1) HIV infection or severe immunodeficiency; (2) malignancy with disseminated disease; (3) insufficient sample volume for mNGS or failed sequencing; (4) incomplete medical records.

Tuberculous pleurisy (TBP) was diagnosed based on a comprehensive evaluation integrating clinical features, pleural fluid characteristics, molecular findings, and response to anti-tuberculosis therapy. In accordance with national and international TB guidelines, patients were classified as having TBP when pleural biopsy demonstrated granulomatous inflammation compatible with TB, or when pleural fluid or pleural tissue tested positive for TB-DNA using nucleic acid amplification tests. Patients presenting with a unilateral lymphocyte-predominant exudative pleural effusion accompanied by elevated ADA or a positive T-SPOT, after exclusion of malignancy, bacterial empyema, and autoimmune diseases, were also considered highly suggestive of TBP. In addition, cases without an alternative diagnosis after routine evaluation but showing clear clinical or radiologic improvement following anti-TB treatment during follow-up were classified as clinically diagnosed TBP. All TBP diagnoses were confirmed by experienced respiratory and infectious disease specialists.

**Figure 1:**
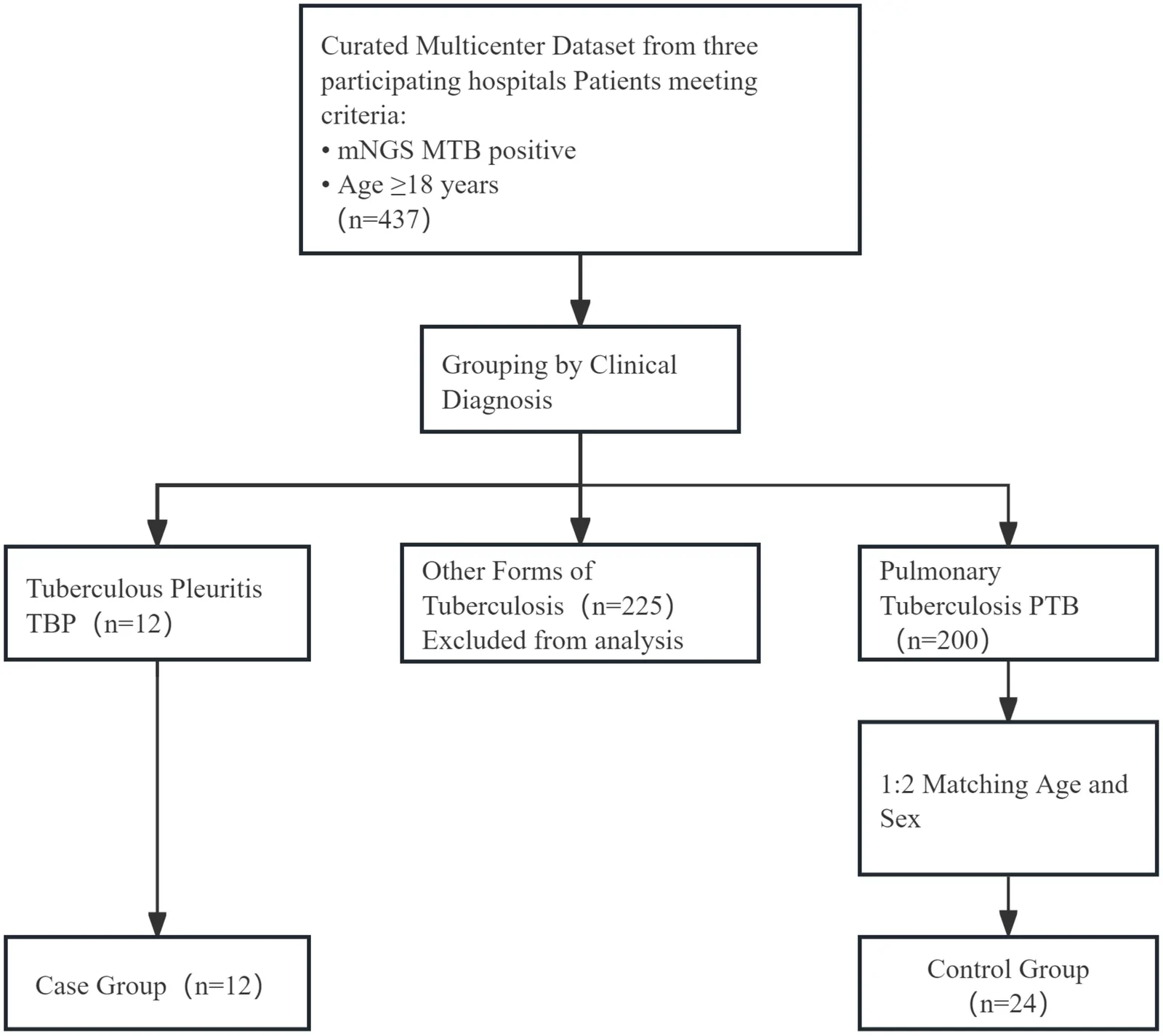
Flowchart of patient selection and study design. This flowchart illustrates the patient selection process from the initial multicenter dataset to the final analytical cohort. Patients with other forms of tuberculosis were excluded from the current analysis. The 1:2 matching was based on age and sex. Patients with immunosuppressive conditions were excluded from both groups prior to matching.

Among 437 eligible patients, 12 cases with tuberculous pleurisy (TBP) were identified as the case group. Pulmonary tuberculosis (PTB) patients were matched at a 1:2 ratio based on age and sex, yielding a final cohort of 36 patients. Patients with HIV infection, active malignancy with disseminated disease, or requiring systemic immunosuppressive therapy were excluded from both groups prior to matching; therefore, immunosuppressive status was uniformly absent across all included patients and is not reported as a separate variable in **Table 1**. (**Figure 1**). Clinical outcomes were determined based on both discharge status and follow-up information, and categorized as favorable (cured or continuing anti-tuberculosis treatment) or unfavorable (death, loss to follow-up, or persistent symptoms with discontinuation of therapy).

**Table 1:** Baseline characteristics of patients.

Variable	Case (n = 12)	Control (n = 24)	P value
Demographics			
Age, IQR (years)	51.0 (29.0-67.8)	49.5 (30.0-70.8)	0.070
Male sex, n (%)	8.0 (66.7%)	15.0 (62.5%)	1.000
mNGS and laboratory indicators, median (IQR)			
MTB read counts	1976.5 (515.5 - 9678.0)	990.0 (278.8 - 27944.8)	0.920
WBC (×10^9^/L)	7.32 (4.88 - 9.23)	5.97 (4.32 - 7.68)	0.179
Neutrophils count (×10^9^/L)	5.10 (2.98 - 7.63)	3.88 (2.69 - 5.14)	0.107
Lymphocytes count (×10^9^/L)	0.91 (0.60 - 1.63)	1.32 (0.89 - 1.75)	0.298
CRP (mg/L)	39.53 (23.00 - 68.73)	17.00 (1.27 - 57.00)	0.058
PCT (ng/mL)	0.07 (0.06 - 0.33)	0.07 (0.04 - 0.17)	0.494
IL-6 (pg/mL)	34.21 (19.18 - 39.00)	20.35 (12.35 - 59.21)	0.671
ESR (mm/h)	54.00 (24.50 - 101.50)	15.50 (8.75 - 38.75)	0.101
Ferritin (ng/mL)	599.75 (399.60 - N/A)	192.05 (135.70 - N/A)	0.3121
Clinical symptom, n(%)			
Fever	7 (58.3%)	11 (45.8%)	0.480
Chest tightness	4 (33.3%)	6 (25.0%)	0.700
Dyspnea	1 (8.3%)	3 (12.5%)	1.000
Headache	0 (0%)	1 (4.2%)	1.000
Cough	8 (66.7%)	11 (45.8%)	0.302
Fatigue	3 (25%)	4 (16.7%)	0.664
Specimen type, n (%)			
Bronchoalveolar Lavage Fluid	3 (25.0%)	21 (87.5%)	0.007
Pleural Source (Pleural Fluid/Tissue)	5 (41.7%)	1 (4.2%)	–
Other	4 (33.3%)	2 (8.3%)	–

Data are presented as median (interquartile range, IQR) or number (%). The Mann-Whitney U test was used for continuous variables, and the Fisher’s exact test was used for categorical variables. A dash (-) indicates that the P value is not repeated as it pertains to a different category of the same variable already tested. Abbreviations: MTB, Mycobacterium tuberculosis; WBC, White blood cell; CRP, C-reactive protein; PCT, Procalcitonin; IL-6, Interleukin-6; ESR, Erythrocyte sedimentation rate.

This study was approved by the institutional review boards of all participating centers (Approval No. 2025-KY-1148-002). Owing to its retrospective nature, informed consent was waived. All data were anonymized prior to analysis.

### Data collection

Clinical data were retrospectively collected from the electronic medical record system, encompassing: (1) demographic characteristics (age and sex); (2) clinical manifestations (major symptoms and key imaging features such as granuloma or necrosis); (3) laboratory parameters (lymphocyte count, neutrophil count, C-reactive protein, and erythrocyte sedimentation rate); and (4) results of tuberculosis-specific assays (T-SPOT.TB, TB-DNA, molecular identification of mycobacterial species, and acid-fast bacilli smear). All enrolled patients completed follow-up, with clinical outcomes categorized as either favorable (cured or still undergoing anti-tuberculosis treatment at last follow-up) or unfavorable (death, loss to follow-up, or persistent symptoms with discontinuation of therapy). For the TBP subgroup prognostic analysis, patients still on treatment at follow-up were reclassified as having an unfavorable short-term outcome, as they had not yet achieved treatment completion; this reclassification and its rationale are described in the relevant Results section.

### Sample types and preprocessing

A total of 36 clinical specimens were analyzed, including bronchoalveolar lavage fluid (BALF, n = 24), pleural effusion (n = 4), tissue samples (lung or pleural tissue, n = 5), drainage fluid (n = 2), and pus (n = 1). All samples were collected under sterile conditions and transported on ice to the microbiology laboratories. BALF and other fluid samples were centrifuged to concentrate microbial components, whereas tissue samples were homogenized using sterile bead-beating tubes. All centers followed harmonized sample-handling procedures.

### Metagenomic next-generation sequencing workflow

Nucleic Acid Extraction:Total nucleic acids (DNA + RNA) were extracted using unified protocols across all centers. Extracted nucleic acids were quantified using spectrophotometry, and RNA integrity was confirmed through automated electrophoresis. Quality control criteria were standardized across centers to ensure reproducibility.

Library Preparation and Sequencing: Sequencing libraries were prepared through mechanical fragmentation, end-repair, adapter ligation, and purification. Libraries passing quality thresholds were sequenced on Illumina high-throughput platforms according to the manufacturers’ instructions. Each batch contained negative (no-template) and positive controls.

Bioinformatic Analysis: Raw reads underwent quality trimming, removal of adapter sequences, and subtraction of human reads (aligned to hg38). Non-human reads were aligned to curated microbial genomes and clinically validated pathogen reference databases. Reads assigned to the M. tuberculosis complex were manually reviewed for depth and genomic coverage. All centers employed standardized bioinformatic pipelines and predefined filtering thresholds (Chiu and Miller, 2019; Miller et al., 2019; Gu et al., 2021).

### Definition of MTB positivity by mNGS

Samples were considered MTB-positive if they met all of the following criteria: at least one high-quality read uniquely aligned to the Mycobacterium tuberculosis complex was detected, no corresponding reads were present in the negative control of the same sequencing batch, and the genome coverage pattern of the detected reads was consistent with MTB reference sequences. This definition is consistent with previously published clinical mNGS guidelines.

### Conventional tuberculosis-related tests

T-SPOT.TB Assay:Peripheral blood mononuclear cells were stimulated with ESAT-6 and CFP-10 antigens according to the manufacturer’s instructions. IFN-γ–producing spot-forming units were quantified by ELISPOT. A positive result was defined as ≥6 spots in either antigen well and at least twice the negative control, with ≤10 spots in the negative control (King et al., 2015).

Molecular Identification of Mycobacterial Species**:**When required, species identification was conducted via PCR amplification and sequencing of 16S rRNA or rpoB genes.

TB-DNA Detection:Commercial PCR-fluorescence probe kits were used to detect MTB complex DNA qualitatively, based on amplification curves.

Acid-Fast Bacilli (AFB) Smear Microscopy:Specimens were stained using the Ziehl–Neelsen method and examined microscopically. Results were reported semi-quantitatively (1+ to 4+) (Lönnroth et al., 2015).

### ROC curve and AUC analysis

To evaluate the discriminative performance of different clinical and laboratory indicators for differentiating tuberculous pleurisy (TBP) from pulmonary tuberculosis (PTB), ROC curves were constructed and the area under the curve (AUC) calculated. AUC for individual predictors was used to assess their independent discriminative ability (Hajian-Tilaki, 2013); the corresponding P value for each AUC was derived from the Mann–Whitney U test comparing predictor values between TBP and PTB groups, equivalent to testing whether AUC differs from 0.5. Comparisons between single predictors and the combined model were performed using DeLong’s test, and sensitivity and specificity at the optimal Youden index cutoff were reported. The overall model significance was assessed using the likelihood ratio test. A two-sided P < 0.05 was considered statistically significant.

### Statistical analysis

All analyses were performed using SPSS 27 and R 4.2. Non-normally distributed continuous variables were summarized as median (IQR) and compared using the Mann–Whitney U test, while categorical variables were compared using Fisher’s exact test or the chi-square test.

Because mtb_reads showed a skewed distribution, log10(reads + 1) transformation was applied before analysis. Laboratory values reported as below the detection limit (e.g., CRP “<0.5 mg/L”, PCT “<0.02 ng/mL”) were substituted with half of the reported threshold value for statistical analysis, a standard approach for left-censored data. Variables with P < 0.10 in univariate analysis or considered clinically relevant were included in multivariable logistic regression, with ORs and 95% CIs reported. Owing to the small sample size (12 TBP cases, 4 predictors in the model), the event-per-variable ratio (EPV) was approximately 3, well below the commonly recommended minimum of 10. The logistic model is therefore subject to overfitting risk, and all model-derived estimates should be interpreted as exploratory (Jeon et al., 2023). Predicted probabilities from the models were used to generate ROC curves and calculate AUC, with comparisons performed using DeLong’s test. A two-sided P < 0.05 was considered statistically significant.

## Results

### Baseline characteristics and group comparison

A total of 36 patients were included in the final analysis, comprising 12 patients with tuberculous pleurisy (TBP) and 24 with pulmonary tuberculosis (PTB). The baseline characteristics of the two groups are summarized in **Table 1**.

No significant differences were observed in demographic characteristics between groups, including age (median: 51.0 vs. 49.5 years, P = 0.070) and sex distribution (male: 34.8% vs. 65.2%, P = 1.000), indicating good comparability.

Regarding microbiological and laboratory indicators, the Mycobacterium tuberculosis sequence read counts (mtb_reads) did not differ significantly between the TBP and PTB groups (median: 1976.5 vs. 990.0, P = 0.920). Similarly, inflammatory markers—including white blood cell count, neutrophil count, lymphocyte count, procalcitonin, and interleukin-6—showed no significant intergroup differences (all P > 0.05). C-reactive protein (CRP) levels tended to be higher in the TBP group (median 39.53 mg/L vs. 17.00 mg/L, P = 0.058).

A significant difference was observed in the distribution of specimen types between groups (P = 0.007). In the PTB group, bronchoalveolar lavage fluid (BALF) was the predominant specimen type (21/24, 87.5%), whereas in the TBP group, specimens were more heterogeneous: BALF accounted for 25.0% (3/12), pleural-origin specimens (pleural effusion or pleural tissue) for 41.7% (5/12), and other specimen types (drainage fluid, lung tissue, pus) for the remaining 33.3% (4/12). Pleural-origin specimens were significantly more common in TBP than PTB (41.7% vs. 4.2%; Fisher’s exact test, P = 0.007).Regarding clinical symptoms, cough and fever were the most common manifestations in both groups. No significant differences were observed between the TBP and PTB groups in the prevalence of fever (58.3% vs. 45.8%, P = 0.480), chest tightness (33.3% vs. 25.0%, P = 0.700), dyspnea (8.3% vs. 12.5%, P = 1.000), headache (0% vs. 4.2%, P = 1.000), cough (66.7% vs. 45.8%, P = 0.302), or fatigue (25.0% vs. 16.7%, P = 0.664).

### Comparison of mNGS and conventional diagnostic findings in TBP and PTB

**Figure 2:**
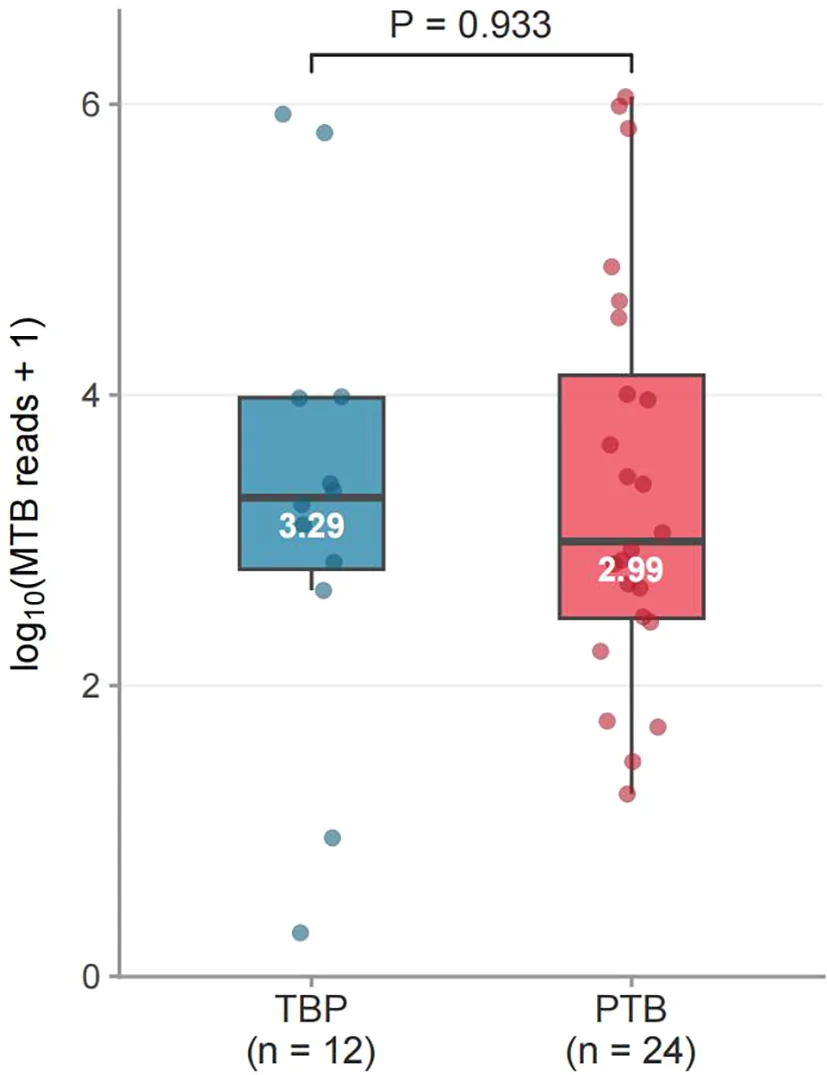
MTB read counts in TBP vs PTB.

The mNGS characteristics are presented in **Table 2**. No significant difference was found in the median MTB read counts between TBP and PTB (1976.5 [IQR: 515.5–9678.0] vs. 990.0 [IQR: 278.8–27944.8], P = 0.920). The box plot (**Figure 2**) showed a broader distribution and higher upper outliers in the PTB group, whereas the TBP group exhibited a more concentrated distribution.

**Table 2:** Comparison of mNGS characteristics between TBP and PTB.

Variable	Case (n=12)	Control (n=24)	P value
MTB read counts [median (IQR)]	1976.5 (515.5–9678.0)	990.0 (278.8–27944.8)	0.920
Mixed infection (n, %)	8 (66.7%)	14 (58.3%)	0.727

Although conventional tests (AFB smear, Molecular identification of mycobacteria, TB-DNA) demonstrated low positivity in TBP patients, all included cases were detected as MTB-positive by mNGS, reflecting the superior sensitivity of mNGS in samples with very low bacterial loads. The rates of co-infection were comparable between groups (66.7% vs. 58.3%, P = 0.727).

The positivity rates of conventional diagnostic methods are shown in **Table 3**. T-SPOT.TB positivity was similar between TBP and PTB (66.7% vs. 63.2%), as were TB-DNA (both 50.0%) and AFB smear results (22.2% vs. 21.1%), with no significant differences. Molecular identification of mycobacteria was performed only in PTB, yielding a positivity rate of 66.7% (6/9), reflecting the suitability of this method for respiratory samples with higher bacterial burdens.

**Table 3:** Comparison of positivity rates of conventional detection methods between TBP and PTB groups.

Detection method	TBP positive/total (%)	PTB positive/total (%)	P value
T-SPOT.TB	6/9 (66.7)	12/19 (63.2)	1.000
TB-DNA	2/4 (50.0)	1/2 (50.0)	1.000
AFB smear	2/9 (22.2)	4/19 (21.1)	1.000
Molecular identification of mycobacteria	0/0 (-)	6/9 (66.7)	–

In this retrospective cohort, none of the patients in the TBP group had undergone molecular identification of mycobacterial species; thus, data were unavailable.

Importantly, all patients in this study tested positive by mNGS (100% positivity rate), which reflects the study’s inclusion criteria rather than an estimate of true mNGS diagnostic sensitivity in unselected patients. Due to the absence of mNGS-negative cases, agreement metrics (e.g., κ statistics) could not be calculated. Within this mNGS-confirmed cohort, conventional methods showed substantially lower positivity rates in TBP (e.g., AFB smear positivity 22.2%), consistent with the paucibacillary nature of pleural disease.

### Diagnostic performance of clinical predictors and the combined model

**Figure 3:**
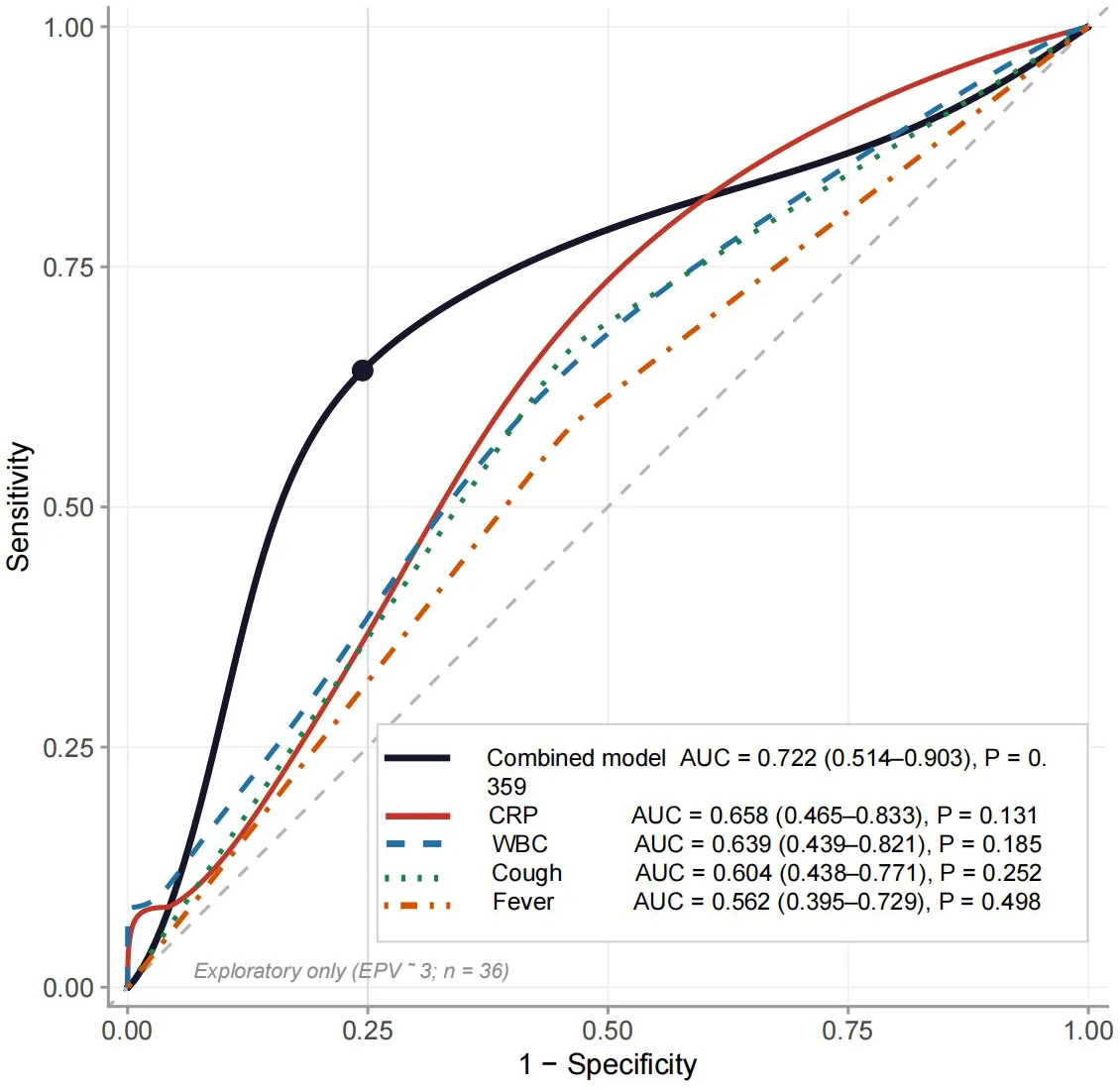
Diagnostic performance of clinical predictors for differentiating tuberculous pleurisy (TBP) from pulmonary tuberculosis (PTB).Receiver operating characteristic (ROC) curves for individual clinical predictors and the combined logistic regression model. AUC values: CRP = 0.658 (P = 0.131), WBC = 0.639 (P = 0.185), Cough = 0.604 (P = 0.252), Fever = 0.562 (P = 0.498). None of the individual predictors reached statistical significance. The combined model (Fever + Cough + WBC + CRP) achieved AUC = 0.722 (overall model likelihood ratio test P = 0.359; sensitivity = 0.667, specificity = 0.833 at optimal Youden index). Given the small sample size and EPV ≈ 3, all estimates should be interpreted as exploratory.

In the ROC analysis, individual clinical predictors showed limited and variable discriminatory ability for differentiating TBP from PTB (**Figure 3**). Among them, C-reactive protein (CRP) demonstrated the highest discriminatory value (AUC = 0.658, 95% CI: 0.443–0.827, P = 0.131), while white blood cell count (WBC) showed a similar trend (AUC = 0.639, 95% CI: 0.428–0.831, P = 0.185). Cough (AUC = 0.604, 95% CI: 0.432–0.777, P = 0.252) and fever (AUC = 0.562, 95% CI: 0.385–0.740, P = 0.498) showed minimal discriminative power. None of the individual predictors reached statistical significance, consistent with the limited sample size and modest effect sizes.

To improve discriminatory performance, a multivariable logistic regression model incorporating cough, fever, CRP, and WBC was constructed (**Table 4** — see below). The regression coefficients, odds ratios (ORs), and 95% confidence intervals for each predictor are presented in **Table 4**. None of the individual predictors reached statistical significance in the multivariable model (all P > 0.25), likely reflecting the limited statistical power with EPV ≈ 3. The combined model yielded an AUC of 0.722 (95% CI: 0.539–0.926, overall model likelihood ratio test P = 0.359), with a sensitivity of 66.7% and specificity of 83.3% at the optimal Youden index threshold. Although the combined model numerically outperformed any single predictor, the non-significant overall model test and small EPV indicate that these results should be interpreted as preliminary only. Prognostic Factors in the TBP Group.

**Table 4:** Multivariable logistic regression for differentiating TBP from PTB variable.

Variable	β	OR	95%CI	P value
Fever	0.543	1.721	0.377-7.851	0.483
Cough	0.704	2.021	0.368-11.104	0.418
CRP (mg/L)	0.007	1.007	0.995-1.020	0.257
WBC (×10^9^/L)	0.133	1.142	0.855–1.527	0.368

Model AUC = 0.722; LR χ² P = 0.359; n = 36; EPV ≈ 3.TBP (n=12) vs PTB (n=24). None of the individual predictors reached statistical significance. The model is exploratory only; overfitting is expected given EPV ≈ 3. External validation is required.

A subgroup analysis was conducted within the TBP group (n=12) to explore factors associated with clinical outcomes. Among the 12 patients, 9 (75.0%) experienced favorable outcomes, while 3 (25.0%) had unfavorable outcomes.

Given that all TBP patients had outcome codes of 0 (cured, n = 9) or 1 (still on treatment at last follow-up, n = 3), patients still on treatment were classified as having an unfavorable short-term outcome for this exploratory analysis, as they had not yet reached treatment completion. This classification should be interpreted cautiously, as “still on treatment” may ultimately lead to cure (**Table 5**). No laboratory, microbiological, or imaging feature reached statistical significance as a predictor of short-term outcome. A non-significant trend toward lower lymphocyte counts was observed in patients still on treatment compared with those who were cured (median: 0.60 vs. 1.10 ×10^9^/L, P = 0.115). This analysis is severely underpowered (n = 12, 3 events) and should be regarded as purely hypothesis-generating. In contrast, white blood cell count, C-reactive protein levels, and mNGS-derived MTB read counts showed no significant differences between the two groups (all P > 0.4).

**Table 5:** Prognostic factors analysis in tuberculous pleuritis patients (n=12).

Characteristic	Favorable outcome (n=9)	Unfavorable outcome (n=3)	P value
Laboratory indicators, median (IQR)			
WBC (×10^9^/L)	7.40 (5.25 - 10.05)	7.24 (3.71 - 7.42)	0.486
Neutrophils count (×10^9^/L)	5.10 (3.45 - 8.30)	6.14 (2.45 - 6.23)	0.937
Lymphocytes count (×10^9^/L)	1.10 (0.71 - 1.80)	0.60 (0.53 - 0.75)	0.115
CRP (mg/L)	36.27 (19.55 - 67.69)	45.39 (35.62 - 255.66)	0.485
PCT (ng/mL)	0.087 (0.060 - 0.540)	0.057 (0.051 - 0.063)	0.225
IL-6 (pg/mL)	31.13 (1.50 - 39.67)	26.25 (15.20 - 37.30)	0.937
Microbiological testing, median (IQR)			
mNGS (mtb_reads)	1761.00 (579.00 - 9625.00)	2455.00 (8.00 - 636530.00)	0.937
Imaging features, n (%)			
Nodule	6 (66.7%)	2 (66.7%)	1.000
Cavity	1 (11.1%)	1 (33.3%)	0.455
Granuloma	4 (44.4%)	1 (33.3%)	0.524
Necrosis	2 (22.2%)	1 (33.3%)	1.000

Data are presented as median (interquartile range, IQR) for continuous variables and n (%) for categorical variables. Group comparisons were performed using the Mann–Whitney U test for continuous variables and Fisher’s exact test for categorical variables. Favorable outcome (n = 9): patients documented as cured at follow-up (outcome code 0). Unfavorable outcome (n = 3): patients still on anti-tuberculosis treatment at last follow-up (outcome code 1), reclassified as unfavorable for this subgroup analysis as they had not achieved treatment completion. No patients experienced death, loss to follow-up, or symptomatic discontinuation of therapy in this cohort. This analysis is severely underpowered (3 events) and all findings should be interpreted as exploratory only.

These findings should be interpreted with caution due to the small number of unfavorable outcomes and require validation in larger prospective studies.

### Association between mNGS findings and the diagnosis of tuberculous pleurisy

Among patients who tested positive for MTB by mNGS, several detection characteristics showed descriptive associations with the clinical diagnosis of TBP. All TBP cases were identified as MTB-positive by mNGS (100%), however, as this reflects the study’s inclusion criteria rather than an estimate of true diagnostic sensitivity, this finding should not be interpreted as evidence of mNGS performance in unselected patients. Conventional diagnostic methods (such as TB-DNA and AFB smear) showed substantially lower positivity rates in the TBP group (≤ 50%), consistent with the paucibacillary nature of pleural disease. Although MTB read counts did not differ significantly between TBP and PTB (1976.5 vs. 990.0, P = 0.920), pleural-derived samples consistently yielded positive mNGS results in this mNGS-confirmed cohort.

TBP patients exhibited a trend toward higher CRP levels compared with PTB (39.53 vs. 17.00 mg/L, P = 0.058), while single mNGS quantitative metrics—such as MTB read counts—did not independently discriminate between the two groups. An exploratory combined model incorporating clinical variables (fever, cough, WBC, CRP) demonstrated improved but non-significant discriminatory ability (AUC = 0.722, overall model P = 0.359), suggesting that integrating routine clinical indicators with mNGS findings may warrant further investigation in adequately powered studies.

## Discussion

This multicenter study evaluated the discriminatory value of mNGS and routine clinical indicators for differentiating TBP from PTB in a cohort restricted to mNGS-confirmed MTB cases. Within this selected population, conventional diagnostic methods exhibited substantially lower positivity rates than mNGS in TBP patients, consistent with prior reports of reduced sensitivity in paucibacillary specimens (Huang et al., 2023; Liu et al., 2025). Importantly, the 100% mNGS positivity rate in our cohort reflects the study design — inclusion was contingent on mNGS positivity — rather than an estimate of the true diagnostic sensitivity of mNGS in unselected TBP-suspected patients; the latter cannot be inferred from our data. Notably, among mNGS-positive cases, pleural-origin specimens were consistently represented in TBP, underscoring the importance of directly sampling the affected compartment when mNGS testing is pursued.

A key observation was the comparable MTB read counts between TBP and PTB (median 1976.5 vs. 990.0, P = 0.920), despite the typically lower viable bacterial load expected in pleural specimens. Several mechanisms may account for this unexpected finding. First, mNGS detects both cell-associated and extracellular DNA, and pleural effusions in TBP may contain substantial quantities of MTB-derived cell-free DNA released through immune-mediated bacterial killing, contributing disproportionately to read counts relative to viable organisms. Second, the diverse specimen types in the TBP group (pleural effusion, pleural tissue, drainage fluid) may have varying DNA extraction efficiencies, potentially inflating read counts in some cases. Third, PTB specimens from cavitary lesions, while having higher viable bacterial burden, may yield more variable and inconsistent read distributions due to sampling heterogeneity within the airways, as reflected by the wider interquartile range observed in our PTB group. These observations collectively suggest that mNGS read counts in pleural specimens should not be interpreted as a direct quantitative surrogate for viable bacterial load, and that specimen type must be considered when comparing mNGS metrics across disease compartments. The wider distribution and presence of high outliers in PTB specimens may reflect greater heterogeneity in bacterial burden within cavitary pulmonary lesions.

Although both TBP and PTB are caused by MTB, they represent distinct clinical-pathological entities. In our cohort, C-reactive protein (CRP) and white blood cell (WBC) counts tended to be higher in TBP patients, which may reflect a more pronounced local inflammatory response within the pleural space (Garcia-Pachon et al., 2005). However, individual clinical or laboratory parameters exhibited limited discriminatory power, suggesting that no single metric can effectively differentiate between these two complex disease states (Choi et al., 2016).

To improve discriminative performance, we constructed a multivariable logistic regression model incorporating cough, fever, CRP, and WBC. Although the combined model yielded a numerically higher AUC (0.722) compared with individual predictors, the overall model did not reach statistical significance (likelihood ratio test P = 0.359), and none of the individual coefficients were statistically significant. Critically, with only 12 TBP cases and 4 predictors (EPV ≈ 3), this model is highly susceptible to overfitting, and the reported AUC is likely optimistically biased. The model should not be regarded as clinically validated or actionable without replication in an independent, adequately powered cohort. Nevertheless, the direction of effects — higher CRP, higher WBC, and presence of cough or fever tending toward TBP — is biologically plausible and consistent with the more pronounced local inflammatory response in pleural disease. This provides a conceptual framework for future, adequately powered studies to test whether such a composite approach can meaningfully distinguish TBP from PTB (Liu et al., 2022; Zhang et al., 2025).

The exploratory prognostic analysis within the TBP subgroup (n = 12, 3 events) was severely underpowered and should be interpreted accordingly. No laboratory, microbiological, or imaging parameter reached statistical significance. A non-significant trend toward lower lymphocyte counts in patients still on treatment versus those who achieved cure (median 0.60 vs. 1.10 ×10^9^/L, P = 0.115) is noted solely as a signal for future investigation; it carries no diagnostic or prognostic weight in the current dataset. The hypothesis that host immune status, as reflected by lymphocyte count, may modulate disease trajectory in TBP is biologically plausible and merits evaluation in adequately powered prospective cohorts.

Strengths of this study include its multicenter design with standardized mNGS laboratory and bioinformatics pipelines, which enhanced technical comparability, and the use of matched PTB controls to reduce confounding by age and sex. Patients with immunosuppressive conditions were uniformly excluded prior to enrollment, ensuring a relatively homogeneous host immune background across both groups. Several important limitations must be acknowledged. First and foremost, the small sample size (12 TBP, 24 PTB) represents a critical constraint. All statistical findings — including AUC values, odds ratios, and P values — should be considered preliminary and hypothesis-generating, not definitive. The study was not powered to detect clinically meaningful differences in most comparisons, and the risk of both Type I and Type II errors is elevated. Second, the logistic regression model incorporated 4 predictors with only 12 cases (EPV ≈ 3), far below the recommended minimum EPV of 10. The model is subject to substantial overfitting, and reported performance metrics are likely optimistically biased. External validation in an independent cohort is required before any clinical application. Third, the inclusion of only mNGS-positive patients introduces a fundamental selection bias: the 100% mNGS positivity rate is an artifact of the inclusion criteria and cannot be used to infer the sensitivity of mNGS for MTB detection in unselected TBP-suspected patients. Clinicians should not apply these findings to decisions about whether to order mNGS. Fourth, direct comparison of MTB read counts between pleural and pulmonary specimens is potentially confounded by differences in specimen biology, cellular composition, DNA extraction efficiency, and sampling technique. Fifth, the retrospective design and reliance on electronic medical records may introduce information bias, and the small number of unfavorable outcomes in the TBP subgroup (n = 3) renders the prognostic analysis essentially uninformative beyond signal generation.

In conclusion, mNGS offers distinct advantages in the diagnosis of TBP, particularly in cases likely to be missed by conventional methods. However, neither mNGS read counts nor individual clinical parameters are sufficient to reliably distinguish TBP from PTB. A combined model integrating multiple clinical and inflammatory markers demonstrated improved discriminative performance, suggesting that incorporating routine clinical indicators can optimize the diagnostic workflow for mNGS-positive patients. Future studies should validate and refine such models in larger prospective cohorts and explore the prognostic value of combining mNGS data with host immune markers. As mNGS becomes more widely adopted, such integrated data strategies will be crucial for advancing precision medicine in tuberculosis care.

## Data Availability

The original contributions presented in the study are included in the article/supplementary material. Further inquiries can be directed to the corresponding author.
